# Assessing Panic: Bridging the Gap Between Fundamental Mechanisms and Daily Life Experience

**DOI:** 10.3389/fnins.2018.00785

**Published:** 2018-10-24

**Authors:** Nicole K. Leibold, Koen R. Schruers

**Affiliations:** ^1^Department of Psychiatry and Neuropsychology, School for Mental Health and Neuroscience, European Graduate School of Neuroscience, Maastricht University, Maastricht, Netherlands; ^2^Faculty of Psychology, Center for Experimental and Learning Psychology, University of Leuven, Leuven, Belgium

**Keywords:** panic attacks, CO_2_ exposure, genetics, DNA methylation, ambulatory assessment

## Abstract

Panic disorder (PD) is one of the most common psychiatric disorders. Recurrent, unexpected panic attacks (PAs) are the primary symptom and strongly impact patients’ quality of life. Clinical manifestations are very heterogeneous between patients, emphasizing the need for a dimensional classification integrating various aspects of neurobiological and psychological circuits in line with the Research Domain Criteria (RDoC) proposed by the US National Institute of Mental Health. To go beyond data that can be collected in the daily clinical situation, experimental panic provocation is widely used, which has led to important insights into involved brain regions and systems. Genetic variants can determine the sensitivity to experimental models such as carbon dioxide (CO_2_) exposure and can increase the risk to develop PD. Recent developments now allow to better assess the dynamic course of PAs outside the laboratory in patients’ natural environment. This can provide novel insights into the underlying mechanisms and the influence of environmental factors that can alter gene regulation by changing DNA methylation. In this mini review, we discuss assessment of PAs in the clinic, in the laboratory using CO_2_ exposure, genetic associations, and the benefits of real-life assessment and epigenetic research.

## Introduction

Panic attacks (PA) are periods of intense fear concomitant with other symptoms such as breathing difficulties and palpitations. PAs are most commonly associated with panic disorder (PD), but are also severity specifiers for all mental disorders in the Diagnostic and Statistical Manual of Mental disorders (DSM-5) (American Psychiatric Association, 2013). Currently, diagnoses of psychiatric disorders are based on the presence of symptoms specified in the DSM. Individuals with very heterogeneous clinical manifestations can meet the required minimum number of symptoms for a disorder and thus receive the same diagnosis and treatment. However, the same treatment might not be equally efficient in these patients. The National Institute of Mental Health [NIMH] (2010) initiated a new research framework, the Research Domain Criteria (RDoC), to classify mental disorders based on dimensions of dysfunction in neurobiological and psychological circuits. By integrating diverse approaches such as molecular circuits and genetics, the translation of fundamental findings to the clinic can be facilitated. It can lead to a better understanding of which functions underlie a specific behavior or symptom, and what degree of dysregulation is associated with a shift from mental health to subclinical presentation and, ultimately, full disorder level. Eventually, this could support establishing a graded classification that opens new approaches for more precise treatment strategies. This mini review provides an overview of methodologies used to assess PAs in the clinic and in the laboratory focusing on carbon dioxide (CO_2_) exposure, and discusses added value and approaches for real-life assessment and epigenetic research.

## Panic Attacks in the Clinic

The diagnostic criteria for PAs and PD are specified in the DSM (American Psychiatric Association, 2013) and serve as guideline for clinicians. Currently, PAs are defined as sudden episodes of intense fear or discomfort, reaching a peak within minutes and accompanied by at least four of 13 symptoms: palpitations, sweating, trembling or shaking, breathlessness, feeling of choking, chest pain, nausea, dizziness, chills or heat sensations, paresthesia, derealization or depersonalization, fear of losing control or going crazy, and fear of dying. PD is characterized by recurrent unexpected PAs, followed by at least 1 month of persistent concerns about the occurrence of future attacks or their consequences, and/or strong maladaptive behavioral changes related to the attacks such as avoiding places and situations. Assessment of these symptoms and behavior is classically based on patients’ self-reports (interviews and rating scales covering presence and intensity of symptoms, frequency of PAs, impairments in daily life), sometimes completed by clinicians’ behavioral observations. Furthermore, a complete psychiatric and medical examination are done to exclude that PAs occur due to, e.g., specific phobia, substance abuse or another medical condition such as thyroid dysfunction. Additional assessment of family history, recent life events, and stress can provide a glance at factors associated with PAs and PD. However, this is insufficient to increase our understanding of molecular alterations and disturbances that make someone vulnerable to develop disorders.

## Panic Attacks in the Laboratory

### CO_2_ Exposure as Experimental Model

To gain insights into the pathophysiology of PAs many studies have made use of experimental panic provocation. Among the many agents used in the laboratory particularly CO_2_ exposure is one of the best validated and most widely used models (Leibold et al., 2015). Commonly, CO_2_ is administered through a nasal-oral facemask and participants rate their symptoms using questionnaires. Particularly 35% CO_2_ effectively induces DSM specified symptoms (Griez, 1984) that are in striking resemblance to real-life PA ones (Griez et al., 1987; Schruers et al., 2004), indicating that an acute disturbance of the acid-base homeostasis might be the mechanism underlying PAs as we previously proposed (Esquivel et al., 2009). PD patients react significantly stronger to CO_2_ than patients of many other disorders (Verburg et al., 1994; Perna et al., 1999; Kent et al., 2001) with the exception of post-traumatic stress disorder patients who are also reactive to CO_2_ (Muhtz et al., 2011; Kellner et al., 2018). Additionally, social anxiety disorder patients show an intermediate reactivity between healthy individuals and PD patients (Schutters et al., 2012). This suggests that some altered mechanisms are common among these disorders.

#### Physiology of Panic Attacks

Given the pronounced physiological symptoms of PAs, research has aimed at obtaining detailed insights into bodily changes during attacks. Breathing 35% CO_2_ led to an increase in heart rate in some studies (Poonai et al., 2000; Richey et al., 2010), while others observed an overall decrease (Argyropoulos et al., 2002; Kaye et al., 2004; Wetherell et al., 2006). General autonomic arousal is suggested by strong increases in systolic (Wetherell et al., 2006; Richey et al., 2010; Leibold et al., 2013) and diastolic blood pressure (Richey et al., 2010; Leibold et al., 2013). High blood pressure can activate a negative feedback loop, the baroreflex, which causes heart rate to decrease to subsequently lead to a reduction in blood pressure. This might explain the reported heart rate reduction in some studies. Some studies reported increases in heart rate; these varying results might be due to differences in methodology (temporal resolution, age and sex of participants, averaging time points).

CO_2_ is a major activator of breathing in mammals and exerts its effects mainly through activation of central chemoreceptors (Rassovsky and Kushner, 2003). Inhaling 35% CO_2_ strongly increased minute ventilation and tidal volume (Bystritsky and Shapiro, 1992). No difference in the degree of increase could be found between PD patients and healthy individuals, which, however, could have been masked by relatively long analysis epochs of 15 s. Regarding breathing, a distinct respiratory subtype was described that is characterized by a high number of respiratory symptoms during PAs and a higher response to CO_2_ (Freire and Nardi, 2012). Non-medicated patients also had a higher variability in respiration rate and partial CO_2_ pressure compared to medicated patients and healthy individuals in the 10 min after the inhalation. Sample sizes were small, but these results might be indicative of a less effective homeostatic control in response to a stimulus such as CO_2_ that acutely disturbs the pH balance in PD patients (Niccolai et al., 2008).

### Neurobiology of Panic Attacks

In recent years, the field of neurobiology has made significant progress to unravel brain regions and networks involved in PD. PD is classified as anxiety disorder, but research indicates that anxiety, fear, and panic are distinct entities. In a seminal human functional imaging study (Mobbs et al., 2007), individuals underwent a virtual predator paradigm. At a large distance, brain activation was mostly observed in the prefrontal cortex, which is associated with complex risk assessment and approach behavior. With decreasing distance brain activity shifted to the brainstem, a subcortical region associated with faster and more primitive responses such as fight-or-flight behavior. The predominant emotions that can be mapped to this “defensive distance” to a threat are anxiety and fear, respectively (Blanchard and Blanchard, 1990; McNaughton and Corr, 2004). Extending this model, conceptually, the smallest possible distance to a threat is one coming from within the body.

A threat from within the body can be caused by an acute disbalance in the pH homeostasis. CO_2_ is a stimulus that can cause such a brief disturbance. We propose that this triggers panic and is primarily coupled to activation of the primordial brainstem. If PAs are linked to primordial brain regions and fundamental mechanisms, it can be expected that every individual reacts to CO_2_. In line with this, we showed that also healthy individuals experience the fear and symptoms associated with PAs, when the dosage is increased compared to PD patients (Schruers et al., 2011; Leibold et al., 2013). This suggests that PD patients are hypersensitive to CO_2_. It remains to be determined which basic mechanism shifts physiological CO_2_-sensitivity to pathological as in PD. A starting point is provided by a functional imaging study by our group (Goossens et al., 2014); in line with our proposition of the involvement of primordial brain regions, we demonstrated that CO_2_ activates the brainstem, in PD to a greater extent than in healthy participants. This indicates that brainstem abnormalities might underlie the hyperreactivity to CO_2_ in patients. Previously, the amygdala was proposed as center of the network involved in PD (Gorman et al., 2000), and in determining fear behavior to CO_2_ in animal work (Ziemann et al., 2009). However, this concept recently got challenged by research in Urbach-Wiethe patients, who unexpectedly experienced experimental PAs to a CO_2_ inhalation despite having a bilaterally damaged amygdala (Feinstein et al., 2013). Our study provides additional evidence that other brain regions such as the brainstem could have a key role. The question remains which neurotransmitter systems and molecules are involved in detecting and reacting to CO_2_/pH changes. Among the many neurons sensitive to CO_2_/pH (Dean et al., 1990; Mulkey et al., 2004; Richerson, 2004; Williams et al., 2007; Biancardi et al., 2008; Ziemann et al., 2009; da Silva et al., 2010, 2011), in context of PD, obvious candidates are proteins, neurons, and systems related to clinically effective medication. For example, Selective Serotonin Reuptake Inhibitors (SSRIs) target the serotonin transporter (5-HTT), suggesting a role of the 5-HT system in PD. As system functioning is affected by the expression of proteins, genetic research has drawn more attention in recent years.

## The Role of Genetics

A wealth of data suggests a role of the genome in the pathophysiology of PD. Family studies consistently revealed that the risk for PD is considerably increased in first-degree relatives (Crowe et al., 1983; Harris et al., 1983; Noyes et al., 1986; Maier et al., 1993; Mendlewicz et al., 1993; Weissman, 1993; Goldstein et al., 1994). Additionally, the concordance rate for PD is higher for monozygotic, genetically identical twins than for dizygotic twins, suggesting that the disorder is genetically driven and not by shared environmental factors (Torgersen, 1983; Kendler et al., 1993; Skre et al., 1993; Perna et al., 1997). Further, genetics also affects CO_2_-hypersensitivity, as demonstrated by first-degree family member studies, having an intermediate CO_2_ response between patients and unrelated healthy controls (Perna et al., 1995; van Beek and Griez, 2000), and twin studies (Bellodi et al., 1998; Battaglia et al., 2007, 2008; Roberson-Nay et al., 2013).

To determine distinct genes that drive the risk for PD, hypothesis-free association and candidate approaches, the latter based on prior evidence for a role in the disease, have been used. Several hundred genes have been studied to date, extensively reviewed previously (Maron et al., 2010). Here, we very briefly focus on the 5-HT system and the amiloride-sensitive cation channel 2 (ACCN2), for which rodent and human work provided support. A length polymorphism in the promoter region of the 5-HTT, the serotonin transporter gene-linked polymorphic region (5-HTTLPR), affects the 5-HTT expression level (Lesch et al., 1996). The short S-allele has a two- to threefold lower expression of the 5-HTT than the long L-allele, associated with a differential 5-HT signaling. While positive associations were reported regarding differential allele frequencies in patients compared to controls (Maron et al., 2005; Talati et al., 2017), other studies (Hamilton et al., 1999; Watanabe et al., 2017), including a meta-analysis (1,025 patients, 1,568 controls) controlling for ethnicity and comorbid agoraphobia could not confirm any association (Blaya et al., 2007). However, some evidence suggests that the S-allele might be associated with more severe symptom severity (Lonsdorf et al., 2009) and the SS genotype with poor health-related quality of life (Kang et al., 2016). Regarding CO_2_-reactivity, healthy L-allele carriers of the 5-HTTLPR have a heightened fear response (Schmidt et al., 2000), which increases dose-dependently (Schruers et al., 2011). However, in PD patients, CO_2_-reactivity was not affected by genotype (Perna et al., 2004). Yet, in the same study, it was observed that female L-allele carriers did respond better to SSRI treatment. Further, one study reported that no association could be found with the 5-HTTLPR but instead with other variants in the gene encoding the 5-HTT (Strug et al., 2010), suggesting that research should cover the entire gene and not solely focus on the promoter region. Mixed results were also found regarding genes expressing enzymes and receptors related to the 5-HT system. A recent meta-analysis (Howe et al., 2016) reported no association for tryptophan hydroxylase 2 (TPH2), the rate-limiting step in the synthesis of brain 5-HT, the 5HT1a receptor, 5-HT1b receptor, 5-HT2b receptor, and the 5-HT3a receptor. A nominal association was found in females regarding the 5-HT2a receptor and monoamine oxidase A (MAO-A), an enzyme involved in the breakdown of 5-HT, which however did not withstand multiple testing correction.

Furthermore, a role of the ACCN2 system is suggested by both rodent and human work. An association was reported between a variant in the ACCN2 gene and PD, but replication failed in an independent larger sample. However, a very heterogeneous sample with only a subset of PD patients might have masked the effects (Hettema et al., 2008). A later large case-control study reported that a variant was associated with the diagnosis of PD (Smoller et al., 2014). The functional consequences of this variant are unknown; authors speculated that it alters the sensitivity to detect and react to a reduced pH. This is a reasonable speculation as it was shown in rodents that the expressed ion channel is essential for CO_2_-induced fear-related behavior (Ziemann et al., 2009). In further support, we provided evidence that the variant is also associated with a differential response to CO_2_ in patients and healthy individuals (Leibold et al., 2017).

Despite the progress in the past few decades, success in genetic research in anxiety disorders such as PD has proven difficult. Mental disorders like PD are very heterogeneous and complex, which make it necessary to study larger samples. One alternative approach is to focus on endophenotypes such as CO_2_-hyperreactivity or neurobiological responsiveness that are considered to depend on less genes than a complex disorder and can therefore be better linked to genes (Gottesman and Gould, 2003), thereby increasing the chances of detecting links between genetic variants and disease susceptibility.

### Imaging Genetics

Modern neuroimaging techniques allow studying effects of genetic variants on neuronal activation to further examine the pathogenesis of PD.

A common approach is to present pictures with emotional value and to compare induced brain activation, as measured by functional magnetic resonance imaging, to the one caused by viewing neutral stimuli. With regard to the 5-HT system, in a small study with PD patients, no effect of the 5-HTTLPR was found on brain activation in response to fearful or angry facial stimuli (Domschke et al., 2006). However, patients carrying the S-allele had an increased amygdala activation to happy faces. Contrary, in healthy individuals, fearful (Hariri et al., 2002), and aversive stimuli (Heinz et al., 2005) evoked increased amygdala activation in s-allele carriers. In addition, a greater coupling between the amygdala and the ventrolateral prefrontal cortex was found in these individuals, possibly linked to an altered capacity to regulate emotional states (Heinz et al., 2005). Furthermore, in PD patients homozygous for a specific 5-HT1A variant, presentation of fearful faces reduced the activity of the right ventromedial prefrontal cortex, the right orbitofrontal cortex, and the right anterior cingulate cortex (Domschke et al., 2006). No effect on amygdala activation was found. In a classical fear conditioning paradigm, in which repeated pairing of an initially neutral stimulus with an aversive event leads to a fear response to the previously neutral stimulus, PD patients with the “protective” low activity MAO-A allele had an increased anterior cingulate cortex activation to presentation of the paired neutral stimulus during the fear acquisition phase (Reif et al., 2014). In addition, carriers of the low activity allele also benefited more from cognitive behavioral therapy, as shown by a higher response percentage in this group. Regarding ACCN2, in addition to detecting that variants in this gene are associated with PD, an association of the PD-associated allele and bilaterally heightened amygdala volume and activity to visual presentation of emotional faces was also observed in healthy individuals (Smoller et al., 2014).

Overall, these studies suggest that processing emotional stimuli might be affected by genetic variants and that altered activation in distinct brain regions might be linked to PD and the vulnerability to develop the disorder. Success of treatment strategies such as cognitive behavioral therapy could, also in part, depend on these factors. Delineating the mechanisms could lead to improved and more individualized treatments.

## From Laboratory to Real-Life Assessment

### Ambulatory Assessment

A general limitation of experimental models is the laboratory setting that does not reflect the natural environment. The highest ecological validity would be provided by studying PAs in a natural setting, i.e., in real-life outside the laboratory. This so-called ambulatory assessment consists of repeated within-day measurements of an individual’s symptoms, behavior or physiology to monitor dynamic changes over time in relationship to the occurrence of PAs. Often a notification at specified or random time points throughout the day is given, requesting to fill out questions about presence and intensity of symptoms at that or near that moment. This momentary, real-time assessment is believed to reduce retrospective recall bias. However, to date, studies are relatively scarce, which is likely due to technical limitations such as a short battery life and very limited storage capacity in the past.

The conducted ambulatory assessment studies showed that palpitations, dizziness, dyspnea, nausea and sweating are among the most often experienced symptoms of real-life PAs (Margraf et al., 1987; Hoehn-Saric et al., 2004). Of these, dyspnea, palpitations, dizziness and chest pain were the most intense one in another study (Meuret et al., 2011). Comparing ambulatory ratings with retrospective questionnaires and structured diagnostic interviews revealed that patients had a distorted recollection and reported a greater number of symptoms, particularly fear of going crazy, faintness, trembling/shaking, and fear of dying. However, the ranking of symptom frequency remained similar (Margraf et al., 1987).

Spontaneous PAs occurred most often at home (Margraf et al., 1987) or when being with family or alone (Meuret et al., 2011); situational ones most often in a car or public places (Margraf et al., 1987). This can lead to significant behavioral changes. However, unexpectedly, patients with agoraphobia were not found to less often visit public places (Dijkman-Caes et al., 1993). Mean activity levels were higher in patients with a higher number of PAs, but there was no clear pattern if activity increased before or after attacks (Sakamoto et al., 2008). Activity was also significantly higher when patients had no comorbid agoraphobia (Clark et al., 1990).

Given the pronounced physiological symptoms during PAs, attempts have been made to delineate cardio-respiratory changes. Early and newer studies observed an increase in heart rate in some self-reported PAs during 6- (Hoehn-Saric et al., 2004) to 24 h recordings (Barr Taylor et al., 1982; Freedman et al., 1985; Cameron et al., 1987), which could not be attributed to physical activity alone (Barr Taylor et al., 1982). Subjective severity of attacks correlated with heart rate. Contrary, in another study consisting of 175 PAs in 27 patients, heart rate did not increase during spontaneous attacks, but did in anticipation of or during experiencing situational attacks (i.e., in feared situations) (Margraf et al., 1987), which is likely due to the anxiety component. Regarding breathing, PAs were associated with increased tidal volume (Martinez et al., 1996; Meuret et al., 2011), which positively correlated with the level of anxiety and fear of dying (Meuret et al., 2011). Transcutaneous arterial CO_2_ levels were reported to decrease (Hibbert and Pilsbury, 1988), which in a later study was observed in only one out of 24 situationally provoked PAs (Garssen et al., 1996). Furthermore, in a rare case study in a dialysis patient, a spontaneous PA occurred with a strong reduction in arterial CO_2_ and pH increase (Salkovskis et al., 1986). Focusing on PD and trait pathophysiological characteristics as a whole did not reveal any differences between patients and controls (Pfaltz et al., 2009), also not during various levels of activity (Pfaltz et al., 2010), suggesting that changes might be limited to more intense phases.

Considering potential interventions, it is particularly interesting to determine factors that precede the onset of PAs. PD patients seem not to be aware of any symptoms before PAs occur (Kenardy and Taylor, 1999), and no increase in anticipatory anxiety was observed (measurement intervals of at least 2 h) (Helbig-Lang et al., 2012). PA expectancy (morning measurement) was also not associated with a higher likelihood of having a PA (Meuret et al., 2011); it only predicted subsequent anxiety (Rodebaugh et al., 2002). Subdividing PAs into unexpected and expected attacks showed that only expected attacks were preceded by an increased level of danger, anxiety, helplessness and a few symptoms (Kenardy and Taylor, 1999). On the physiological level, strong autonomic irregularities do occur, as early as 47 min before PAs (Meuret et al., 2011). More specifically, heart rate strongly increased the minute before the onset and was positively associated with fear of losing control. Skin temperature already increased in the hour preceding the attack, similarly, to a previous report that only measured the minutes around the attack and also observed an elevation (Freedman et al., 1985). These observations suggest that physiological changes rather than subjective emotions are highly valuable to predict the impending occurrence of attacks and to intervene in the future.

### Environment and Epigenetic Modifications

Increasing evidence suggest that neurotransmitter system functioning, such as of the 5-HT system, could be sensitive to environmental stimuli like stress (Homberg and van den Hove, 2012). A mechanism of how environmental factors could affect system functioning is by altering DNA methylation (Guo et al., 2011a), the best studied type of epigenetic modifications. These modifications are the regulatory interface of genes and vital for normal brain processes, including complex cognitive-affective functioning. Dysfunction can contribute to mental disorders. DNA methylation refers to the covalent binding of a methyl group to a cytosine’s pyrimidine ring at position 5 (5-methylcytosine). This occurs more frequently at cytosines located next to a guanine nucleotide, forming a cytosine-phosphate-guanine (CpG) unit. Methylated CpG sites, which are overrepresented in regulatory promoter regions of genes, generally attract chromatin modifiers, disrupt binding of gene transcription factors and attract proteins that silence gene transcription (Klose and Bird, 2006). DNA methylation is carried out by DNA methyltransferases (DNMTs), families of specific enzymes (Hermann et al., 2004). DNMT1 mainly functions as maintenance transferase to retain the current methylation pattern, while DNMT3a and DNMT3b catalyze *de novo* methylation. The role of the DNMT2 family remains unclear. DNA methylation is fairly stable, yet dynamic to environmental factors (Guo et al., 2011a; Szyf, 2013), suggesting that DNA methylation might be a mechanism of how environment factors affect gene expression (Guo et al., 2011a). Demethylation takes place through several potential repair mechanisms. After oxidation of 5-methylcytosine to 5-hydroxymethylcytosine, subsequent base excision repair pathway (Guo et al., 2011b) or DNMT3a and DNMT3b activity might lead to the removal of the hydroxymethyl group (Chen et al., 2012). Additionally, histone acetylation might actively demethylate DNA (Cervoni and Szyf, 2001). Histones are proteins around which DNA is packed into larger nucleosomes. Acetylation takes place on the lysine residue within the N-terminal amino acid tail of histones, which affects chromatin structure and thereby gene accessibility.

DNA methylation can be affected by genetic variants (Wagner et al., 2014) and environmental stimuli (Guo et al., 2011a; Szyf, 2013). Monozygotic twins who are genetically identical can thus still have different gene expressions and therefore differently functioning biological systems. This emphasizes the need for an integrative analysis of genes, epigenetics, and environment. To date, few epigenetic studies have been done in PD and most of these examined candidate genes. The MAO-A gene was found to be hypomethylated in a mixed sex patient group (Ziegler et al., 2016) and in another study in females (Domschke et al., 2012). In the former study, hypomethylation negatively correlated with PD severity and normalized with effective cognitive behavioral therapy. *In vitro* studies showed that a decreased methylation is associated with an increased MAO-A expression (Checknita et al., 2015). Regarding other neurotransmitter systems, hypomethylation of three CpG sites in the glutamate decarboxylases 1 gene, expressing the rate-limiting enzyme in GABA synthesis, has been found in PD patients (Domschke et al., 2013). The assumed increased GABA level associated with hypomethylation might represent a compensatory mechanism that mediates the effects of negative life events. An additional gene, in which hypomethylation was found in PD patients is the gene expressing the corticotropin releasing hormone receptor 1 (CRHR1a) (Schartner et al., 2017). This receptor is essential in the hypothalamic-pituitary-adrenal axis and also affects stress responses by innervating the locus coeruleus. As some studies reported that the majority of PD patients experienced a major life event in the months before the initial PAs (Uhde et al., 1985), the receptor may be the link between stressful life events, stress responses and the risk for PD. Further, in a small case-control study minor methylation variation was found regarding the norepinephrine transporter gene (SLC6a2) (Bayles et al., 2013). In contrast to these neurotransmitter system-related studies, another research line focuses on the role of the immune system in PD. The transcription factor gene Forkhead-Box-Protein P3 (FoxP3) appeared to be hypermethylated in female PD patients (Prelog et al., 2016). This hypermethylation is assumed to be associated with reduced regulatory T-cells gene transcription, which leads to increased T-cell activation and thus inflammation. This could contribute to the higher rates of inflammatory disorders in PD patients.

Very recently, the first two epigenome-wide association studies (EWAS) were published. Shimada-Sugimoto et al. (2017) found 40 CpG sites that were significantly associated with PD. Most of them were hypomethylated and pathway analysis revealed genes in epidermis development, cell cycle regulation, and lymphocyte activation. Examination of some candidate genes such as MAO-A, GAD1 and SLC6a2 did not show any association with PD. However, smoking and medication were not considered, which are known to affect methylation. The second EWAS study consisted of a larger sample, and an independent replication study (Iurato et al., 2017). In females, after multiple testing correction, a differential methylation was found in the Homo sapiens headcase homolog (HECA) gene, a regulator in the cell cycle and with a potential role in cancer. Its link with PD has yet to be determined. Inclusion of 15 candidate genes showed significant associations with the 5-HT1a receptor in women and 5-HT2a receptor in men, but not GAD1 and CRHR1A as in previous studies. As smoking was not included as potential confounder, these results warrant replication.

## The Need for an Interdisciplinary Approach and Future Perspectives

Experimental panic provocation studies have led to important insights into the nature of PAs in the last few decades. Ambulatory assessment studies are likely to provide further novel knowledge.

Both approaches address questions from different perspectives and bridging the gap between them is expected to open new opportunities, extending our understanding of PAs.

In experimental studies, the controlled environment and immediate effects of CO_2_ to induce PAs allows studying involved fundamental mechanisms in a temporally controlled manner. For example, (epi)genetic research and pharmacological manipulations can determine whether specific genes and neurotransmitter systems play a role in the sensitivity to CO_2_ and thus presumably PAs. This can strongly contribute to develop new and better treatment options. A major advantage of experimental studies compared to other types such as clinical or epidemiological studies is that induced effects are larger accompanied with smaller variation, making smaller samples sufficient to detect relevant effects. While it has been shown that the fear and symptoms triggered by a CO_2_ inhalation are a good representation of a real-life PA (Schruers et al., 2004), a drawback is that attacks do not occur spontaneously in a natural environment but are provoked in a laboratory. The setting, procedure, and presence of the experimenter can affect individuals’ responses, raising the question to which extent findings can be generalized to daily life.

In contrast to looking at acute effects in the laboratory, assessment of naturally occurring PAs in PD patients’ daily lives provides the powerful opportunity to unravel the dynamic course over time. This approach has a higher ecological validity than laboratory models. A drawback is that prolonged recordings are required for comprehensive monitoring, which has been limited by the rather small storage capacity and short battery life of previous devices. The discomfort associated with carrying large setups also restricted sampling to small study samples in the past, but recent advances have led to small devices such as smartwatches that can be worn without being perceived as bothersome. Modern devices also have built-in actigraphy to quantify rest and activity patterns and to record body position, thereby providing more detailed situational data. Incorporating these and other measurements such as physiological parameters, and thereby moving from a main focus on patient ratings (e.g., feelings and symptoms) to automatic assessment, could help to develop interventions targeting the phase preceding attacks and to empower patients to use the device’s feedback for self-directed changes in behavior. This approach puts the individual patient into the center and stimulates informed decisions. Likewise, ambulatory assessment is also suited to test the efficacy of treatments in real life. In the long-term, monitoring after successful treatment could identify individuals at risk to relapse and initiate early prevention. Using advanced devices measuring a broad spectrum of parameters, from physiology to context, can help to determine how symptoms are interrelated and interact with natural environmental factors that are controlled in a laboratory setting.

Environmental factors individuals are exposed to in real-life can affect molecular mechanisms such as epigenetic modifications and thus gene expression. Therefore, it is important to strive for an integrative, interdisciplinary approach of daily-life and fundamental research. For instance, determining which environmental factors are related to the disease and what their functional effects are on a molecular level could provide highly valuable insights into the pathophysiology of PAs. A major challenge is to identify the relevant environmental factors. In this respect, cumulative environmental risk scores combined with genotypes and epigenetic pattern could provide vastly informative data about which interactions are relevant and potentially identify risk groups to develop the disorder. Nowadays it is possible to cost-effectively determine epigenetic pattern in high-resolution, i.e., site-specific DNA methylation. However, these analyses require multiple testing correction for each CpG site, letting many sites fail to reach significance. An approach to tackle this issue could be the development of a novel correction methods similar to the ones applied in genome-wide association studies (Li and Ji, 2005), taking into account the often highly correlated methylation levels of adjacent CpG sites to reduce the number of tests.

Moreover, most epigenetic PD studies to date were association studies, which do not allow drawing any conclusions about the exact role of DNA methylation. One approach to imply a causal role is the two-step Mendelian randomization method, in which first the causal impact of a risk factor on DNA methylation is examined, followed by testing the causal effect of DNA methylation on the outcome (Relton and Davey Smith, 2012). Experimentally, large longitudinal studies can determine whether methylation changes over time are associated with developing the disorder. In this context, rodent models could be a highly beneficial addition as they allow molecular manipulations that exceed ethical possibilities in humans. For instance, studying behavioral consequences to central infusion of pharmacological compounds that target molecules of the epigenetic machinery and thereby lead to overwriting DNA methylation patterns could strengthen causality assumptions (Weaver et al., 2004). At the same time, environmental factors can be controlled to assess their effects. We and others have analyzed rodents’ behavioral performance under CO_2_ exposure (Ziemann et al., 2009; Johnson et al., 2012; Leibold et al., 2016). Adding for example cardio-respiratory monitoring as outcome variables, which can also be done in humans, further enhances the similarity between experiments and increases the translational value (Leibold et al., 2016). We recently provided a quantitative comparison between rodents, healthy volunteers, and PD patients and showed that the physiological response in mice corresponds well to the one in both human groups (Leibold et al., 2016). Such a model overcomes the challenge to align observed behavioral performances in rodents with behavioral self-reports in humans and maximizes translation of data between species. This can significantly drive forward research and eventually application of discoveries. For instance, once causality is suggested, efforts could focus on developing pharmacological compounds targeting epigenetic enzymes to normalize methylation pattern in rodents. Compensatory genes could be activated to reduce symptoms or even obtain a biochemical balance to eventually let patients fully recover. A caveat is that complex disorders are most likely caused by a network disturbance rather than a single gene, but such a starting point might inspire new directions and eventually brings us closer to better treatments.

## Conclusion

The manifestation of PAs can vary widely between people. To better understand the dimensional physiology and pathology and to facilitate a new classification in line with the RDoC project a more interdisciplinary approach of genome × epigenome × environment interactions is needed. Overall, integrating fundamental and real-life research can greatly advance the field, determine biomarkers to identify individuals at risk, and support developing more effective treatment strategies.

## Author Contributions

NL and KS equally contributed to manuscript writing and revision and approved the submitted version.

## Conflict of Interest Statement

The authors declare that the research was conducted in the absence of any commercial or financial relationships that could be construed as a potential conflict of interest.
